# Supplementary figures and images for: Using a Simple Cellular Assay to Map NES Motifs in Cancer-Related Proteins, Gain Insight into CRM1-Mediated NES Export, and Search for NES-Harboring Micropeptides
Source: Int J Mol Sci. 2020 Sep 1;21(17):6341. doi: 10.3390/ijms21176341 (PMC7503480; doi:10.3390/ijms21176341)

Supplementary Figure 1

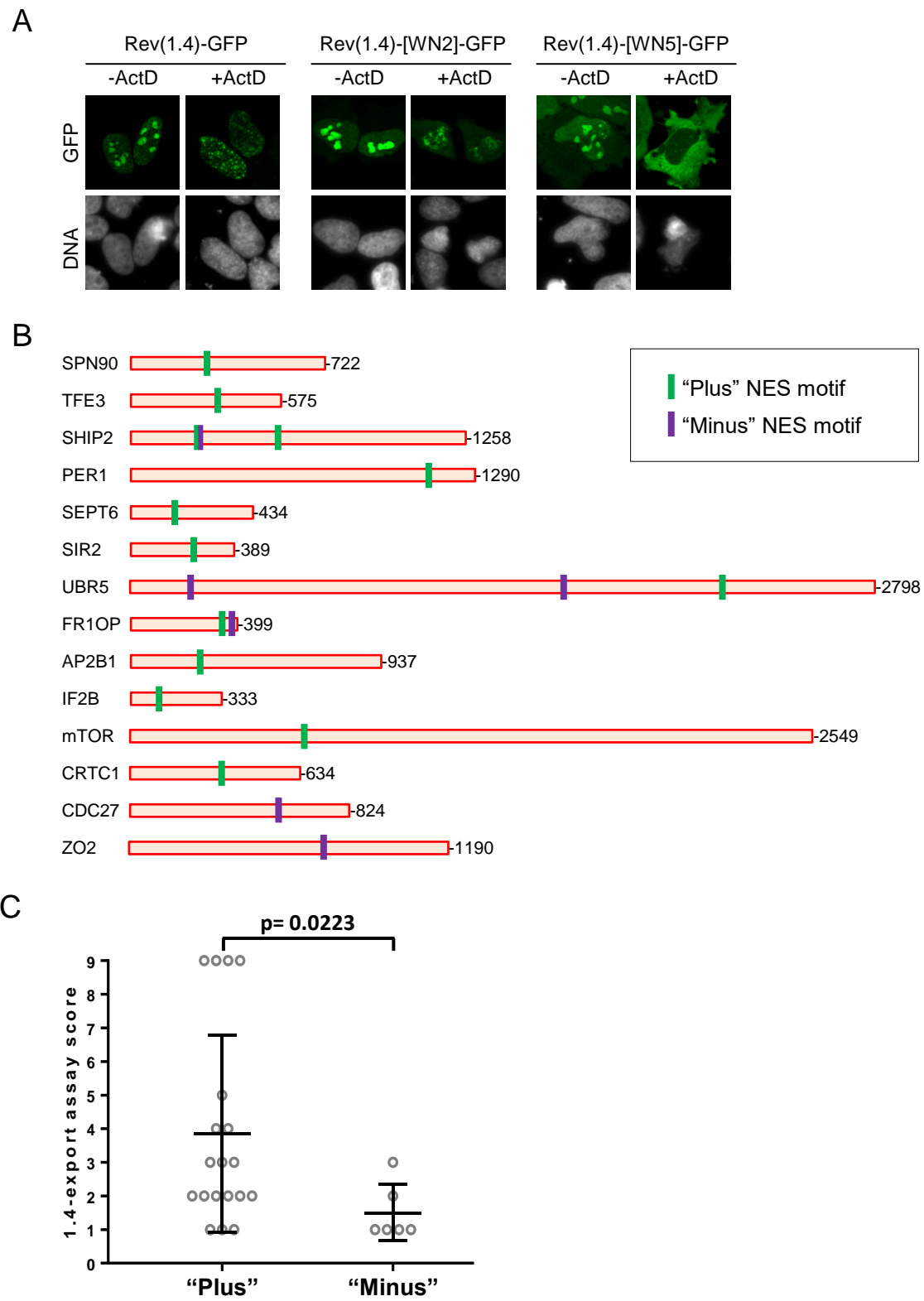

Supplement: Supplementary file 1 [file ijms-21-06341-s001.zip › Sendino et al SuppFigure 1.pdf]

Supplementary Figure 2

Class 1

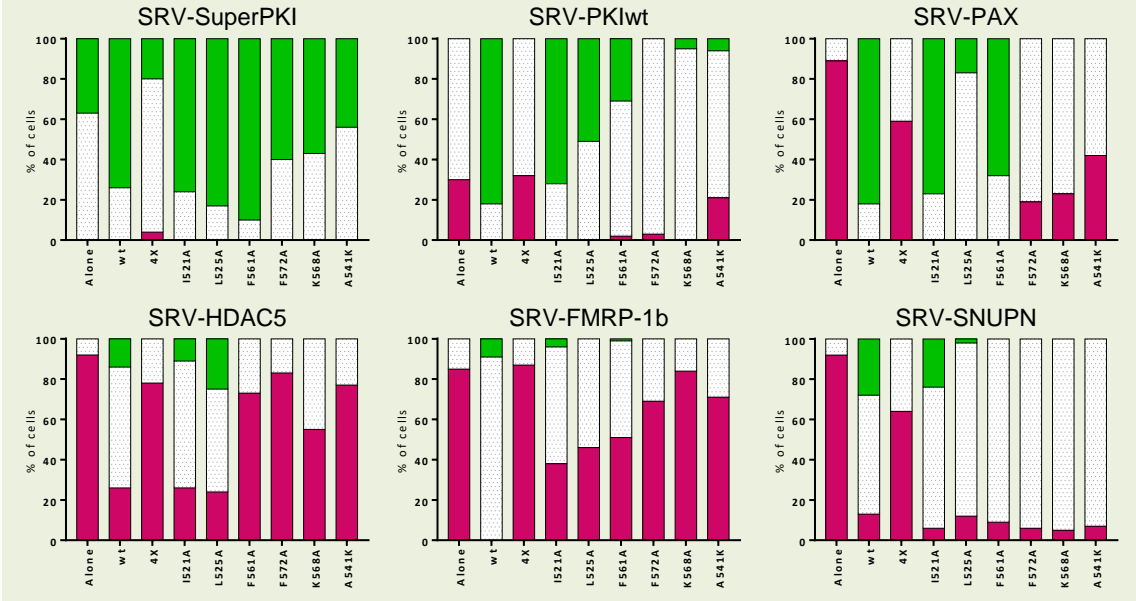

Class 2

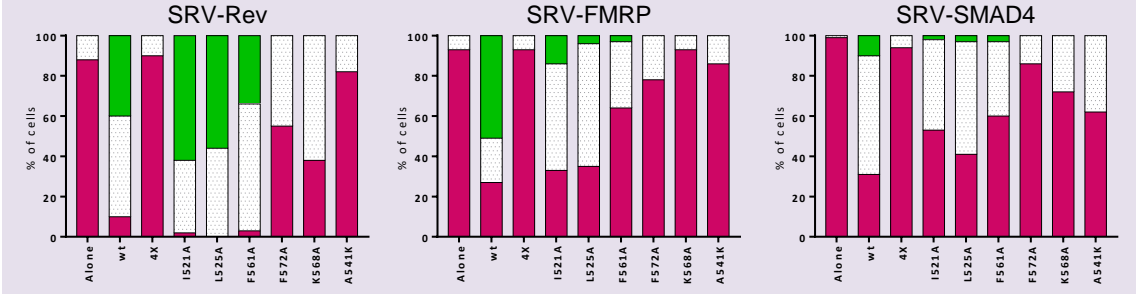

Class 3

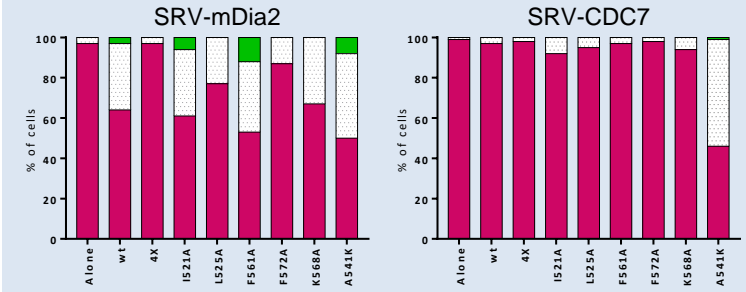

Class 4

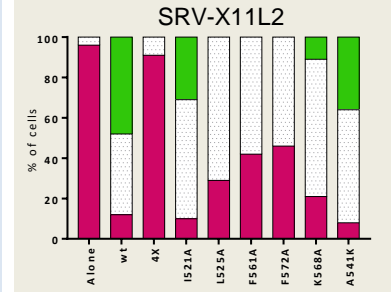

Class 1R

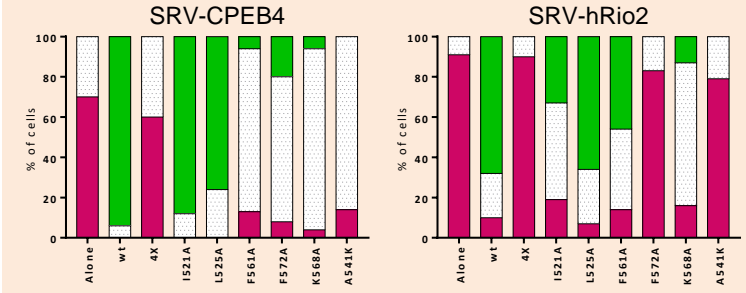

Localization of SRV<sub>B/A</sub> reporters

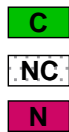

Supplement: Supplementary file 1 [file ijms-21-06341-s001.zip › Sendino et al SuppFigure 2.pdf]

Supplementary Figure 3

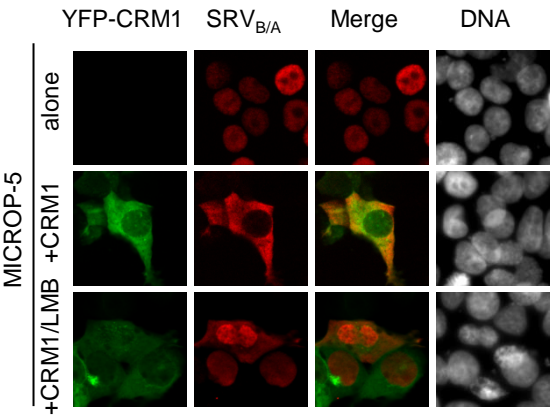

Supplement: Supplementary file 1 [file ijms-21-06341-s001.zip › Sendino et al SuppFigure 3.pdf]
